# Finding Direction in the Search for Selection

**DOI:** 10.1007/s00239-016-9765-5

**Published:** 2016-12-02

**Authors:** Grant Thiltgen, Mario dos Reis, Richard A. Goldstein

**Affiliations:** 1 Institute of Child Health, University College London, London, UK; 2 The School of Biological and Chemical Sciences, Queen Mary University of London, London, UK; 3 Division of Infection & Immunity, University College London, London, UK

**Keywords:** Positive selection, * d*_N_/*d*_S_, Directional selection, Diversifying selection

## Abstract

Tests for positive selection have mostly been developed to look for diversifying selection where change away from the current amino acid is often favorable. However, in many cases we are interested in directional selection where there is a shift toward specific amino acids, resulting in increased fitness in the species. Recently, a few methods have been developed to detect and characterize directional selection on a molecular level. Using the results of evolutionary simulations as well as HIV drug resistance data as models of directional selection, we compare two such methods with each other, as well as against a standard method for detecting diversifying selection. We find that the method to detect diversifying selection also detects directional selection under certain conditions. One method developed for detecting directional selection is powerful and accurate for a wide range of conditions, while the other can generate an excessive number of false positives.

**Electronic supplementary material:**

The online version of this article (doi:10.1007/s00239-016-9765-5) contains supplementary material, which is available to authorized users.

## Introduction

When a new mutation arises in a population, the mutation can be advantageous, deleterious, or effectively neutral, with a sufficiently small effect on the organisms fitness that its fate depends on genetic drift. When organisms are well adapted to their environment, mutations with large advantages are rare. Under these conditions, the deleterious mutations are mostly eliminated from the population while some fraction (~$\frac{1}{2 N_{\rm e}}$ for diploid organisms with effective population size $N_{\rm e}$) of the neutral mutations become fixed, so that most accepted mutations are neutral or nearly neutral (Kimura 1983). This situation is considered *purifying* selection. There are situations, however, where the organisms are not so well adapted. An organism may have changed environments and has to adapt to its new circumstances. A pathogen might have switched host species and needs to adapt to the new host species’ cellular factors. Sometimes new opportunities arise, such as following a gene duplication event, where one of the gene copies can gain a new function while the other maintains its previous function. When such situations occur, there may be a significant possibility of advantageous mutations. The advantageous mutations can be strongly selected for so that the majority of the fixed mutations are adaptive even if most mutations are deleterious or neutral. This situation is called *positive* selection. In the situations mentioned above, where the organism is adapting to a new environment or to new opportunities, the positive selection would be characterized as *directional* selection, as new rare alleles will be favored that better adapt the organism to its new situation. After this process is completed, the organism may become well adapted to its new environment, and purifying selection will resume (dos Reis 2015).

Under certain circumstances, however, this adaptation may never finish, resulting in continued positive selection. An example is the interactions between a pathogen and the immune system of its host. The pathogen will be under strong selection to make mutations that prevent detection from the hosts’ immune system, resulting in fixed mutations that interfere with this detection. Once these mutations are accepted, however, the immune system is under strong selection for mutations that enable the pathogens to be detected. If the host is successful in combating the evasions of the pathogens, the pathogen will once again experience selection for new escape mutations. There is a competition, an arms race, between pathogen and host, where both sides are under selection to counter the changes of the other. This phenomenon, first proposed by Van Valen (1973), was named the “Red Queen Effect” after the character in Lewis Carroll’s *Through the Looking-Glass and What Alice Found There* (Carroll 1871). Situations such as this are called *diversifying* selection, as it is generally the new rare mutants that are selected.

Identification of positive selection can provide important information about a protein’s function, interaction partners, and physiological context, as well as insights into the processes of adaptation, pathogen host shifts, and neo- and sub-functionalization. Of the two types of positive selection described above, directional selection and diversifying selection, it has been easier to detect diversifying selection. Because of the constant selection of advantageous mutations in both host and parasite, there is an elevated rate of fixation of mutations. If we assume that the selection is acting mostly on the expressed proteins rather than directly on the genetic material, this will result in a higher fixation probability for non-synonymous mutations. If synonymous substitution are neutral, we can use the synonymous substitution rate as an internal reference and consider the ratio of the relative rates of non-synonymous (*d*_N_,* K*_a_) and synonymous substitutions (*d*_S_,* K*_s_), a measure referred to as* d*_N_/*d*_S_,* K*_a_/*K*_s_, or $\omega$. When all mutations are neutral (such as in a pseudogene), mutations that change the protein sequence will have the same fixation probability as those that do not,* d*_N_ $\sim$ *d*_S_, and $\omega \sim 1$. Under conditions of purifying selection, mutations that change the amino acid sequence are more likely to be deleterious and thus not accepted, while synonymous mutations would be neutral. As a result,* d*_N_ <* d*_S_, and $\omega < 1$, as is observed for most proteins. If positive selection is sufficiently strong that it dominates the background purifying selection, non-synonymous mutations may actually be more likely to be fixed than synonymous neutral mutations. Under these conditions, it is possible to observe* d*_N_ >* d*_S_, and $\omega > 1$. The first observations of such diversifying selection were in adaptive immune system proteins such as the major histocompatibility complexes (Hughes and Nei 1988, 1989) and immunoglobulins (Tanaka and Nei 1989). These were followed a few years later by similar observations in pathogens (Bonhoeffer et al. 1995; Hughes and Hughes 1995; Endo et al. 1996). The success of these studies resulted in many considering positive selection to be equivalent to diversifying selection.

Many situations, however, do not fit the diversifying selection paradigm. Often we are interested in the process of adaptation where the red queen effect is absent, such as the previously mentioned cases of migrations, environmental shifts, host shifts, and gene duplication. We would expect that these instances of directional selection would result in a burst of non-synonymous substitutions during the interval of adaptation, resulting in an increased value of * d*_N_/*d*_S_. In fact, there are numerous examples where * d*_N_/*d*_S_ ratios have been used to identify periods of directional selection (Endo et al. 1996; Messier and Stewart 1997). (Interestingly, many of these examples involve genes involved in reproduction, where other forms of competition exist). Such approaches are possible when many locations are under sufficiently strong selection on a short branch, which may not reflect common situations (Hughes 2007). One important limitation is that the sites that are under strong directional selection during these periods are also likely to be under strong purifying selection at other times (Messier and Stewart 1997), making it less likely that there would be sufficiently many non-synonymous substitutions during the episodic directional selection to generate* d*_N_/*d*_S_> 1 (dos Reis 2015).

An alternative approach to identifying directional selection is to look for significant changes or differences in the selective constraints. A number of methods have been developed including looking for an overabundance of large changes in physicochemical properties (Woolley et al. 2003) and detecting sustained or transient changes in substitution rates (Knudsen and Miyamoto 2001; Dorman 2007; Creevey and McInerney 2002; Gu 2006). While simultaneously identifying the sites and branches where this change of selection occur is extremely difficult, in many biological investigations (including migrations, environmental shifts, host shifts, and gene duplications) the branch of the phylogenetic tree is known or can be hypothesised; when this is the case, more complicated substition models can be implemented. Methods have been developed that consider changes in the preferences for specific amino acids, such as modifying codon models of diversifying selection to include enhanced substitution rates to prefered amino acids (MEDS) (Pond et al. 2008; Murrell et al. 2012) and implementing a sitewise dependent codon based mutation selection model with explicit changes in selective constraints (swMutSel) (Tamuri et al. 2009; dos Reis et al. 2011; Tamuri et al. 2012). The hope was that approaches specifically designed for detecting specific changes in selection would be more accurate and powerful in these cases than methods designed for diversifying selection such as* d*_N_/*d*_S_. There is evidence that this is true; Murrell et al. compared the ability of MEDS to characterize sites of directional selection in the development of HIV-1 drug resistance compared with a model of episodic diversifying selection; most locations were better fit by the MEDS model (Murrell et al. 2012).

We consider the case where the branch under episodic directional selection is known. We first use simulated data to characterize the relative accuracy and statistical power of a * d*_N_/*d*_S_ ratio method [as implemented in the PAML software package (Yang 2007)] for detecting directional selection, and how that compares with approaches based on detecting and characterizing the changes in selection that accompany directional selection as implemented in swMutSel (Tamuri et al. 2014) and MEDS (Murrell et al. 2012). We then compare the ability of PAML, swMutSel and MEDS to detect directional selection in the development of HIV-1 drug resistance, extending the comparison by Murrell et al. (2012).

## Results

### Comparisons with Simulated Data

**Fig. 1:**
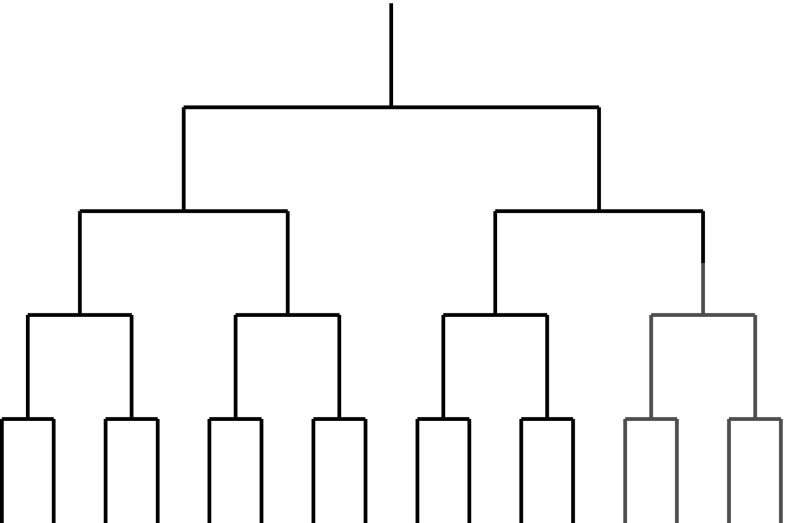
16 taxa. The branches with altered selective constraints are shown in* red*; the change in selection occurs at the midpoint of the branch connecting one quarter of the taxa to the rest of the tree. Data was simulated as described in Tamuri et al. (2012), with a fraction ($\theta$) of the sites in the protein evolving under different selective constraints in the two different parts of the tree. The connecting branch was labeled as the foreground branch in the branch-site analysis of PAML (Yang 2007) (Color figure online)

As discussed in the introduction, although* d*_N_/*d*_S_ methods such as PAML (Yang 2007) were designed to analyse diversifying selection, they are commonly used to detect directional selection. We first created a set of simulated data under a population genetic model where the substitution rate of locations is obtained using probability of fixation under Wright–Fisher mutation-selection model (as described in (Tamuri et al. 2012) and included in swMutSel) and analyzed the ability of PAML (Yang 2007) to detect directional selection. 16 and 256 taxa symmetric bifurcating trees were created, where each branch was the same length *d*, with $d = \{0.01, 0.1, 0.2, 0.5, 1.0\}$ where the values represent the average number of nucleotide substitutions per codon expected under neutral selection (the 16 taxa tree is shown in Fig 1). DNA sequences of 500 codons evolved according to an evolutionary model where most locations evolved under purifying selection, with a fixed percentage of locations ($\theta = \{1, 5, 10, 20\%\}$) undergoing directional selection. This was implemented by having a change of selection at these locations occurring at the midpoint of a specified branch, chosen so that 1/4 of the taxa had the different selective constraints. The first analysis involved modeling these locations as a conserved alanine up to the change of selection, followed by a conserved valine on the downstream branches after this change. This corresponds to an infinitely large shift in selection at this point. (An alanine to valine substitution results from any C to T transition in the second codon position).

The PAML *site model* analyses involve two steps (Nielsen and Yang 1998). The first is the comparison of two models using the likelihood ratio test (LRT), where one model allows the possibility that some sites are evolving with positive selection i.e.,* d*_N_/*d*_S_ $\equiv \omega > 1$, where this condition is assumed to persist throughout the phylogenetic tree. If there is statistical support for the presence of positive selection, a Bayes Empirical Bayes (BEB) analysis is done to identify the corresponding sites. Alternatively, the presence of episodic positive selection is investigated using the “branch-site model” (Yang and Nielsen 2002); the branch on which the change in selection occurs is labeled the “foreground branch”, and the two stage analysis (LRT followed by BEB) is used to find sites that undergo positive selection at this branch, where the model assumes purifying or neutral selection at these sites along the remaining background branches on either side of the foreground branch.

In all of the simulations, the site model analysis resulted in either insufficient support for the presence of positive selection (as indicated with the likelihood ratio test), or in the few cases where such support was present, no sites were identified through the BEB analysis. Because of this, we will restrict our analysis to the results obtained with the branch-site model.

Table 1 shows the results obtained for the 256 taxa tree. PAML was able to identify directional selection when there were a large number of sites undergoing a change in selective along short branches [the positive results required short branches in order to reduce the number of non-synonymous changes occuring under neutral or slight purifying condition (dos Reis 2015)]. Although PAML was able to detect the presence of positive selection when there were long branches with few sites (LRT), PAML was unable to determine which sites were under directional selection (BEB).

**Table 1:** Results of PAML branch-site model analysis of 256 taxa tree with infinite fitness change

d	*θ*			
	0.01	0.05	0.1	0.2
0.01	–	–	(100/0.7)	(100/0.5)
0.1	–	–	–	(100/4.0)
0.2	–	–	–	–
0.5	(0.0/0.0)	–	–	–
1.0	(0.0/1.0)	(0.0/1.0)	–	–

Data sets where PAML could not detect the presence of positive selection using the LRT are shown by a “–”. The percentage of sites undergoing positive selection that were identified, as well as the percentage of other sites that were misidentified, is shown for data sets for which the LRT indicated positive selection

The number of 256-taxa trees that could be studied was limited by computational resources. To see the reproducibility of the data, we also performed analyses of 16 taxa trees, with 100 different sets of simulated data for each set of parameter values, the results of which are shown in Table 2. The results obtained with PAML are similar to the analysis of the 256-taxa trees: PAML can detect when a large number of sites undergo a large change in selective along short branches. In all cases the number of false positives was limited (<5%).

**Table 2:** Results of PAML branch-site model analysis of 16 taxa trees with infinite fitness change

d	*θ*			
	0.01	0.05	0.1	20
0.01	–	–	(87/87/ 0.4)	(100/100/0.4)
0.1	–	–	–	(57/57/2.8)
0.2	–	–	–	(7/7/0.3)
0.5	–	–	–	–
1.0	–	–	–	–

100 identical data sets were created for each set of parameter values. Data sets where PAML could not detect the presence of positive selection with the LRT are shown by a “–”. The percentage of data sets where positive selection was identified, the percentage of sites undergoing positive selection that were identified, as well as the percentage of other sites that were misidentified, is shown where there are data sets for which the LRT indicated positive selection

**Fig. 2:**
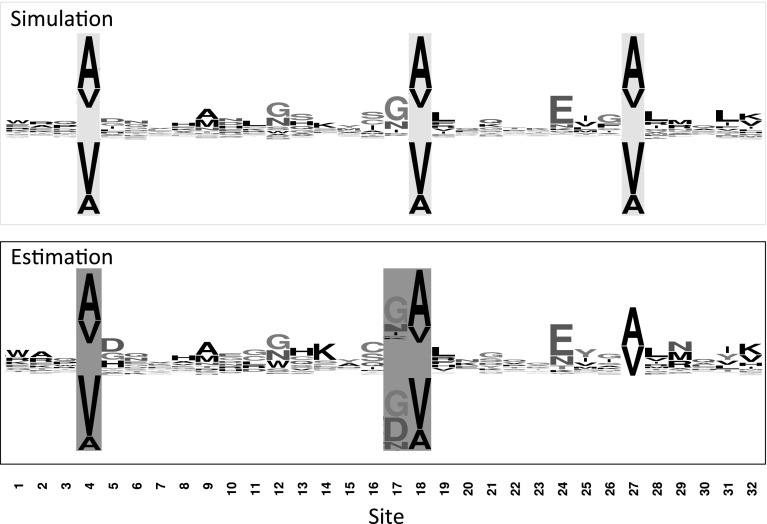
*Top* Sequence logo representing the equilibrium frequencies of the various amino acids at a set of illustrative sites.* Sites 4*,* 18*, and* 27* (highlighted in* yellow*) are simulated with a shift in selective constraints, with $S = 1.0$; equilibrium frequences before and after the change in selection are shown on* top* and* bottom*, respectively.* Bottom* Equilibrium frequencies for these sites as estimated by swMutSel ($d = 0.1$).* Sites* identified as under positive selection highlighted in* blue*; the separate equilibrium frequencies for the branches before and after the change in selection are shown on* top* and* bottom*, respectively.* Sites 4* and* 18* represent true positives,* site 17* a false positive, and* site 27* a false negative. Logos were generated using *WebLogo* (Crooks et al. 2004) (Color figure online)

We next analyzed a set of simulated data, again with 256 taxa symmetric bifurcating trees. (Trees, simulated data, and analysis results are contained in Online Resource 1). Rather than having otherwise conserved amino acids replaced at a specific point on the tree, we instead considered an adjustable degree of directional selection. For a fixed percentage of locations ($\theta = \{1, 5, 10, 20\%\}$), we let the equilibrium frequencies of alanine and valine switch between $\pi _{\rm A} = \frac{\exp \left( S\right) }{1 + \exp \left( S\right) }$, $\pi _{\rm V} = \frac{1}{1 + \exp \left( S\right) }$ and $\pi _{\rm A} = \frac{1}{1 + \exp \left( S\right) }$, $\pi _{\rm V} = \frac{\exp \left( S\right) }{1 + \exp \left( S\right) }$: scaled selective coefficient $S=2N_{{\rm e}}s$, where $N_{\rm e}$ is the effective chromosomal number and *s* is the difference in fitness corresponding to the two amino acids, represents the magnitude of the change of selective pressure, so that $S = 0$ corresponds to no change in selection, with $\pi _{\rm A} = \pi _V = 0.5$ throughout the tree, while $S = 1$ corresponds to a shift from $\pi _{\rm A} = 0.788, \pi _V = 0.212$ to $\pi _A = 0.212, \pi _V = 0.788$. The sequence logo representing the equilibrium frequencies for a set of illustrative sites is shown in Fig. 2.

**Table 3:** Results of PAML branch-site model analysis of 256 taxa tree with fitness changes with a magnitude specified by *S*

d	*S*											
	0	0.1	0.5	1	5	10	0	0.1	0.5	1	5	10
	*θ* = 0.01						*θ* = 0.05					
0.01	–	–	–	–	–	–	–	–	–	–	–	–
0.1	–	–	–	*	–	–	–	–	–	–	–	–
0.2	–	–	–	–	–	–	–	–	–	–	–	–
0.5	*	*	*	*	*	*	*	*	*	*	*	–
1.0	*	*	*	*	*	*	*	*	*	*	*	*

d	*S*											
	0	0.1	0.5	1	5	10	0	0.1	0.5	1	5	10
	*θ* = 0.1						*θ* = 0.2					
0.01	–	–	–	–	–	–	–	–	–	–	–	–
0.1	–	–	–	–	–		–	–	–	–	$\ddag$	$\ddag$
0.2	–	–	*	–	–	–	–	*	*	–	$\ddag$	–
0.5	*	–	–	*	–	–	*	*	–	–	–	–
1.0	*	*	*	*	*	–	*	*	–	–	–	–

($S = 0$ indicates absence of positive selection). Data sets where PAML could not detect the presence of positive selection with the LRT are shown by a “–”. * represents data sets where the LRT indicated the presence of positive selection, but no sites undergoing positive selection were identified. $\ddagger$ indicates data sets where the LRT indicated positive selection, and the BEB correctly identified some of the sites

Running time for PAML for these runs was approximately 4 h on a single core processor; multiple replications are recommended to ensure convergence, resulting in an average running time of approximately 24 h for each dataset. For the datasets with varying fitnesses, the performance of PAML decreased considerably, as is shown in Table 3. There were relatively few cases when the LRT indicated the presence of positive selection, and the occasions when this occurred for the longer branches seem to have been spurious—similar results were obtained with $S = 0$, that is, when positive selection was absent. In these situations, the BEB analysis did not identify specific sites seemingly under positive selection. There were only three conditions were specific sites were correctly identified, corresponding to many locations ($\theta = 0.2$) with strong changes in selection ($S = 5, 10$) along short (but not the shortest) branches. The short branches are required in order for there to be sufficiently few non-synonymous substitutions expected under neutral selection, while the shortest branches were too short for the adaptive substitutions, even when highly advantageous, to take place.

**Fig. 3:**
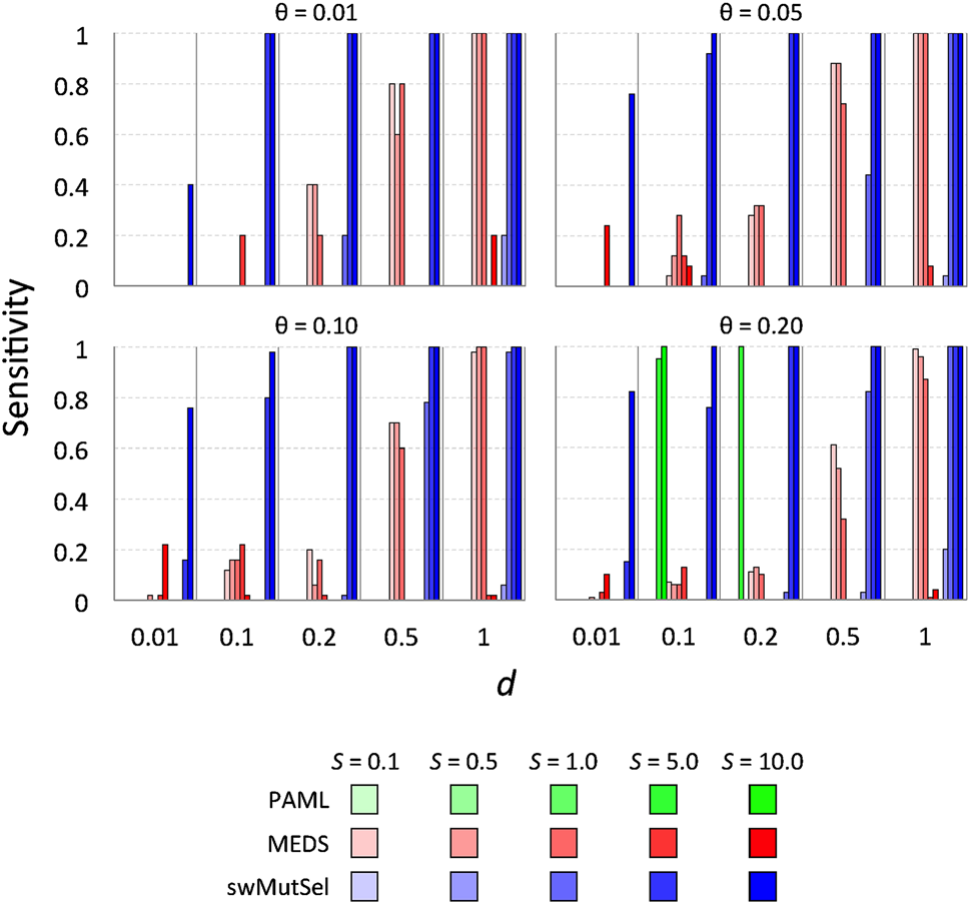
Sensitivity of PAML, MEDS and swMutSel on simulated data. The* green bars* represent the sensitivity (fraction of sites undergoing directional selection correctly detected) of PAML, the* red bars* represent the sensitivity of MEDS and the* blue bars* represent the sensitivity of swMutSel. The range of values are from* light* ($S =$ 0.5) to* dark* ($S =$10). For the PAML results, the sensitivity shown is from the combinations that passed the LRT and the number of correctly predicted sites using the BEB analysis (Color figure online)

We next compared the sensitivity and false positive rate of PAML, MEDS (Pond et al. 2008; Murrell et al. 2012) and swMutSel (Tamuri et al. 2009; dos Reis et al. 2011; Tamuri et al. 2012), the latter two tools constructed specifically to detect directional selection. Both programs are time intensive, with running times of approximately 1 week on a single core processor for each dataset. swMutSel models the selective constraints at each site individually, both assuming uniform equilibrium frequences throughout the tree and separate equilibrium frequencies on either side of the change in selection, using a LRT to evaluate the evidence for a shift in selection. Figure 2 shows the estimated equilibrium frequences for a set of illustrative sites for $S = 10$ and $d = 0.1$.

**Fig. 4:**
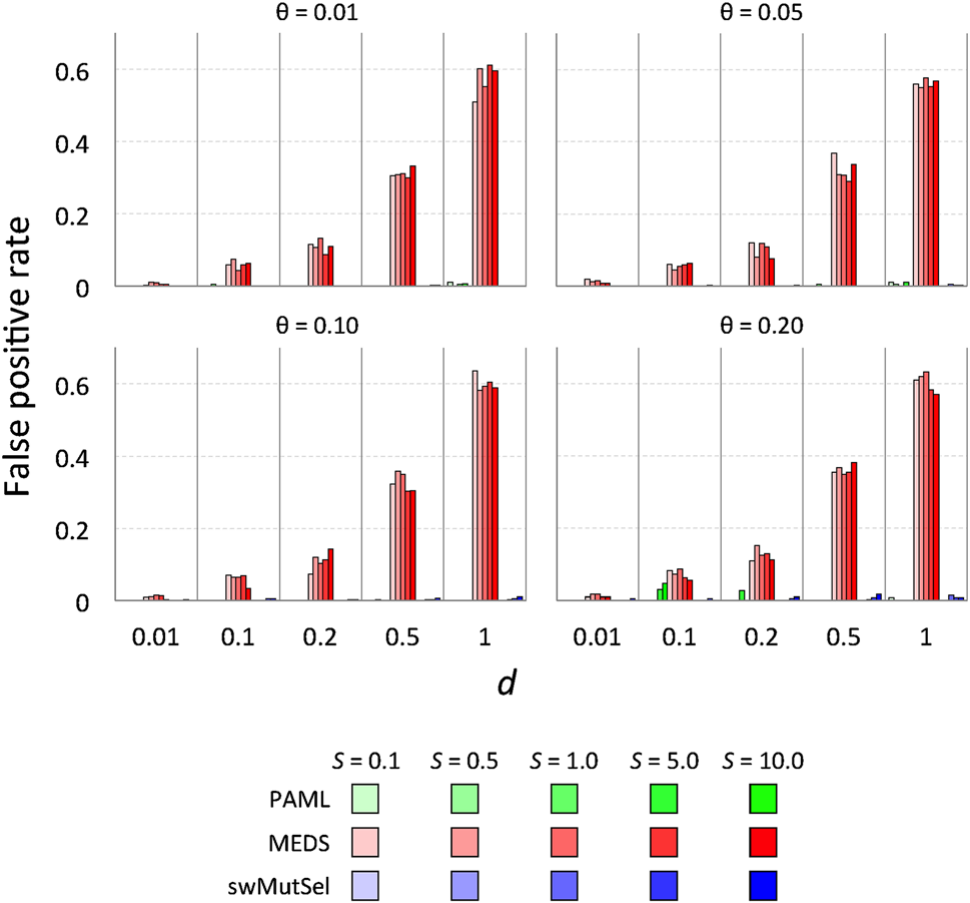
False positive rate of PAML, MEDS and swMutSel on simulated data. The* green bars* represent the false positive rate (fraction of sites undergoing purifying selection misidentified as under positive selection) of PAML, the* red bars* represent the false positive rate of MEDS and the* blue bars* represent the sensitivity of swMutSel.* Colors* and* shades* are as in Fig. 3. For the PAML results, the false positive rates shown are from the combinations that passed the LRT and were misidentified using the BEB analysis. For both with PAML and swMutSel, the false positive rate is quite low (<5%) (Color figure online)

**Fig. 5:**
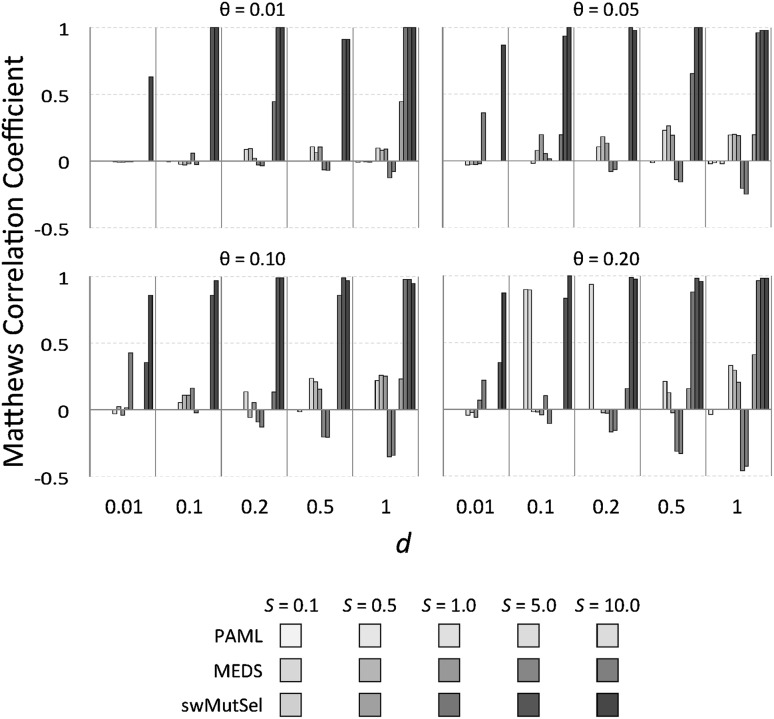
Comparative performance of PAML, MEDS and swMutSel on simulated data, as measured by the Matthews Coefficient (Matthews 1975).* Colors* and* shades* are as in Fig. 3. The Matthews correlation coefficient is equal to one for a perfect predictor and zero for a random predictor, while values less than zero indicates performance worse than random. The poor performance of MEDS is a result of the large false positive rate shown in Fig. 4 (Color figure online)

Figure 3 shows the sensitivity of these three approaches, measured as the number of true positives (TP) divided by the total number of true positives and false negatives (FN) or $\frac{\rm TP}{\rm TP+FN}$. Figure 4 shows the false positive rate for these methods. The overall performance of the three approaches, quantified by the Matthews coefficient (Matthews 1975), is shown in Fig. 5. (Numerical values are available in Supplementary Table S1). PAML was successful at finding many of the correct sites when there were many sites (large $\theta = 0.2$) undergoing large changes in selective constraints (large $S = 5.0, 10.0$) when the branch lengths were small ($d = 0.1, 0.2$). In contrast, swMutSel consistently out-performed PAML, especially when few locations underwent changes in selection (small $\theta$), for longer branch lengths (larger *d*), and under certain conditions, significantly weaker changes in selection (smaller *S*). PAML does have more false positives under the conditions where it has the highest sensitivity (i.e. shorter branch lengths, larger *S* , larger $\theta$), although the false positive rate for both PAML and swMutSel, under all conditions, is quite low (<5%). MEDS seems to identify positive episodic selection in a broad range of parameter values, but with extremely high false positive values (>60%). Overall performance is, as a consequence, poor (Fig. 5).

**Fig. 6:**
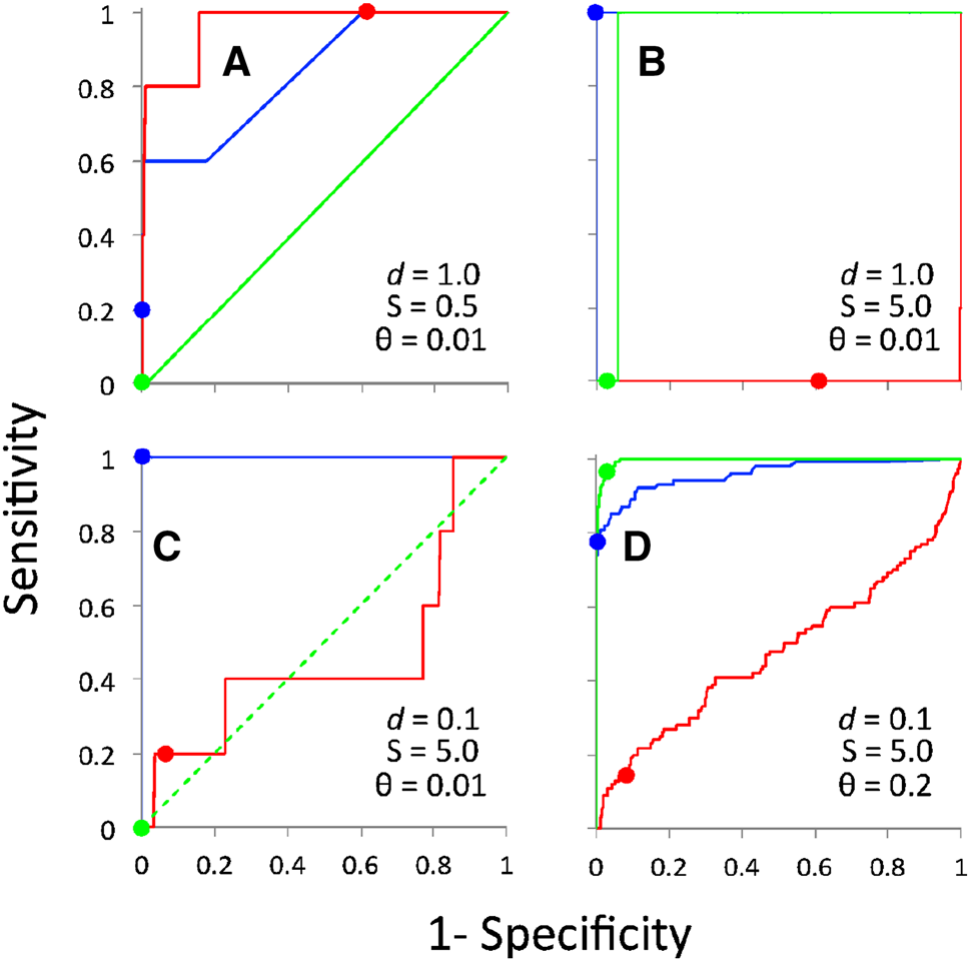
Receiver Operating Characteristic (ROC) plots of PAML (*green*), MEDS (*red*) and swMutSel (*blue*) for illustrative simulations.* Dashed green line* indicates where the data as analysed by PAML did not pass the LRT.* Dots* represent performance for a* p* value of 0.05 (Color figure online)

**Fig. 7:**
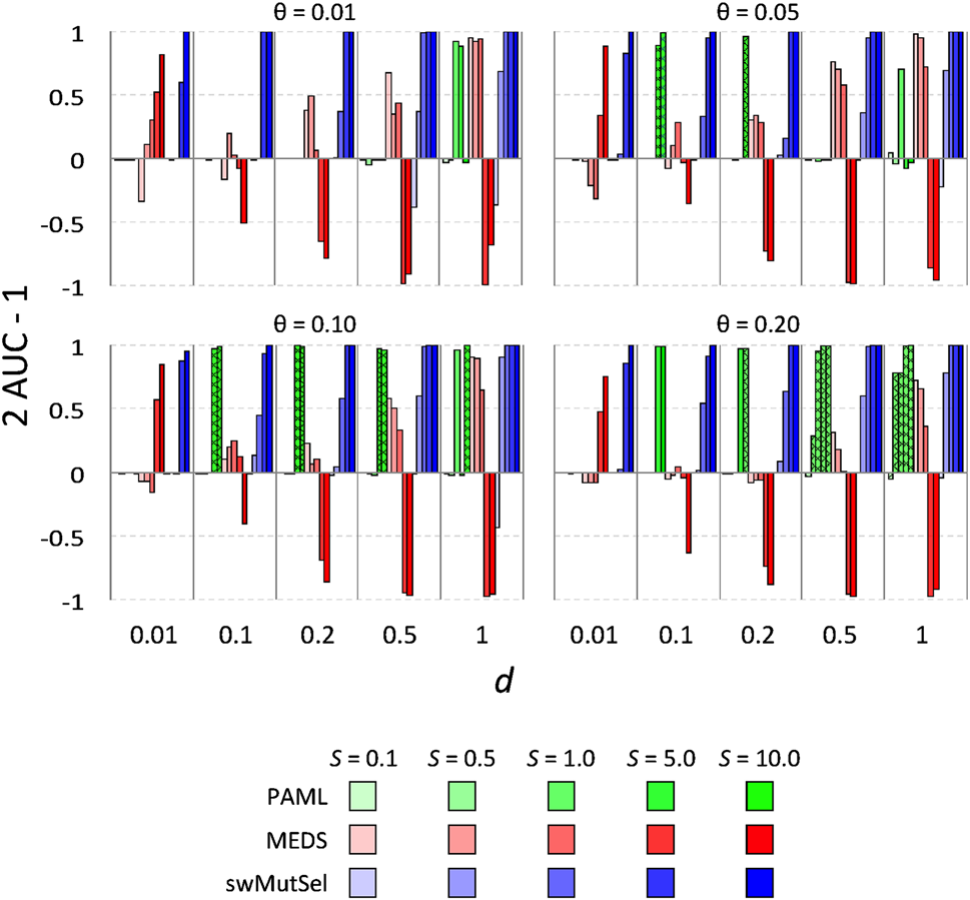
Comparative performance of PAML, MEDS and swMutSel on simulated data, as measured by the 2 AUC − 1, where AUC is the area under the curve of the ROC plot (see Fig. 6).* Colors* and* shades* are as in Fig. 3;* cross-hatching* indicates where PAML indicates inadequate evidence for the existence of positive selection. 2 AUC − 1 is equal to one for a perfect predictor and zero for a random predictor, while values less than zero indicates performance worse than random (Color figure online)

We can investigate the performance further by considering illustrative Receiver Operating Characteristic (ROC) plots for the three approaches, shown in Fig. 6, as well as the area under the ROC curve (AUC) values for all of the simulations shown in Fig. 7. (Numerical values are available in Supplementary Table S1). Consistent with the results presented in Fig. 5, PAML does well for large changes in selection across many sites along a branch that is short but not too short (Fig. 6d). PAML also seems to perform well discriminating between the two types of sites when positive selection is rarer (such as in Fig. 6b), although the conservative choice of* p* value in these cases prevents identification of such sites. MEDS seems to be able to discriminate between sites under and not under directional selection when the difference in selection is very weak (Fig. 6a) and when the branches are short, although the peformance is compromised by overly high* p* values in the former cases. When the change in selection is strong and branch lengths are longer, MEDS often performs substantially worse than random (Fig. 6b). The performance of swMutSel is good to excellent across these various datasets.

**Fig. 8:**
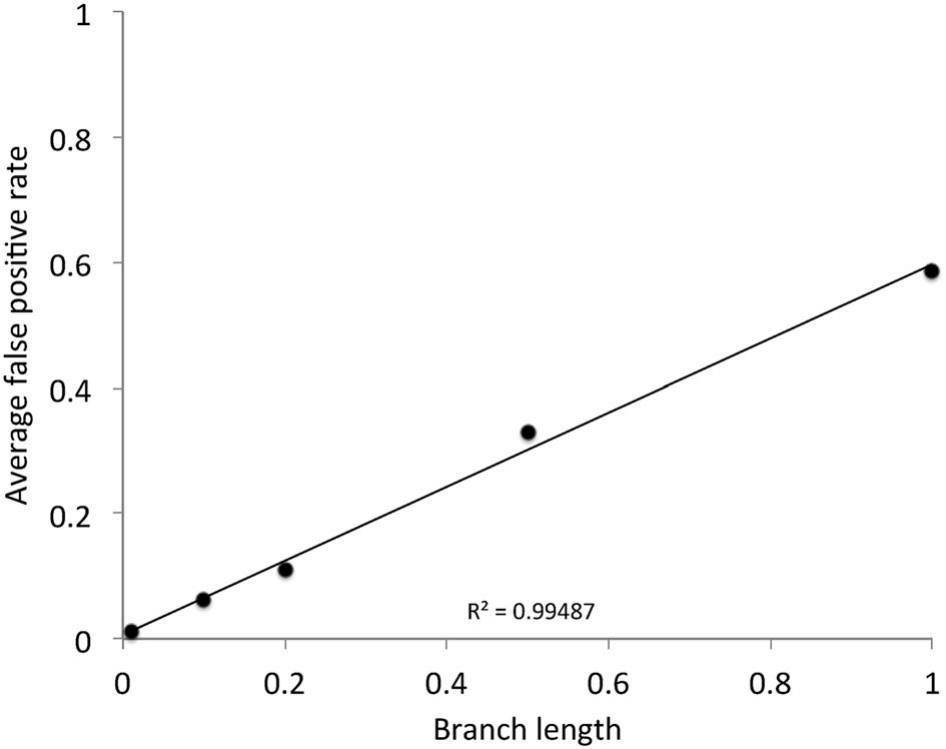
Branch length against the average false positive rate on simulated data. The average was taken over the twenty simulated data sets at each branch length

One noticeable thing about the false positive rate of MEDS is that as the branch length increased, the false positive rate was higher. Figure 8 shows the average false positive rate at each of the branch lengths (*d*), averaged over the twenty simulated data sets. The data indicate a strikingly linear relationship between the average false positive rate and the branch lengths ($R^{2}=0.99487$). This result suggests that MEDS should be restricted to datasets that have very short branch lengths.

### Comparisons with Drug Resistance Data

Simulations are useful for analyzing positive selection detection methods in that the parameters defining the evolutionary process can be specified, and the “correct” answers are known. It is also important to test the methods on real data, even if comparisons may be more difficult and the results more anecdotal. We tested the ability of different approaches to detect sites in HIV undergoing positive selection for drug resistance, a test developed by Murrell et al. (2012). We applied PAML, MEDS, and swMutSel to three viral proteins, protease, integrase, and reverse transcriptase, to see how well they detected the substitutions identified as indicative of drug resistance, while not identifying the locations that are not associated with drug resistance. The results are summarized in Table 4. The specific sites found for protease, integrase and reverse transcriptase can be found in Supplemental Tables S2, S3, and S4, respectively.

All three methods did equally well with the integrase, swMutSel and PAML did equally well on the protease, while PAML did significantly better than either other method on the reverse transcriptase. MEDS did significantly worse than the other methods on the protease. In general, none of the methods had a high rate of false positives; the highest was a 6% rate obtained with MEDS on the protease data set. This indicates that, with a lack of data, the methods tend to be conservative.

**Table 4:** Fraction of sites found by MEDS, PAML and swMutSel for the HIV drug resistance dataset, divided into known sites (K) that have been observed to be affected by drug resistance mutations (Wensing et al. 2014), and unknown sites (*U*) that have not been so identified, as well as the Matthews correlation coefficient (Matthews 1975)

Protein	Length	Number of sequences	PAML			MEDS			swMutSel		
			*K*	*U*	*M*	*K*	*U*	*M*	*K*	*U*	*M*
Protease	99	122	**0.14**	**0**	**0.31**	0.14	0.06	0.19	**0.19**	**0.02**	**0.32**
Integrase	288	295	**0.45**	**0**	**0.67**	**0.45**	**0.004**	**0.61**	**0.45**	**0**	**0.67**
Reverse transcriptase	335	476	**0.52**	**0.007**	**0.65**	0.39	0.02	0.49	0.39	0.01	0.49

The best performing measure for each protein, based on the Matthews correlation coefficient, is shown in bold. (Insignificant differences are ignored). The length and number of sequences used in the analysis has been included

In addition, the locations that are not involved in the development of drug resistance could be under other forms of positive selective pressure, such as compensating for drug resistance mutations. The results are also limited in ways that impact the relative abilities of the various computational approaches.

This is not a perfect test: the drug resistant genes depend upon the specific antiviral drugs taken by the patient, which also varies historically and by location, so it is not clear that a site associated with the development of drug resistance was in fact under directional selection. In addition, there may be selection for drug resistance, or other types of selective pressure, acting on other sites. The development of drug resistance often results in a number of changes at different sites, including changes at some sites that are compensatory for changes at others. This is favorable for those methods (such as PAML) which are sensitive to the number of such sites, as illustrated in the results on synthetic data. There are often a number of possible amino acids substitutions that can occur at a site that assists in drug resistance. For instance, either a K219Q or K219E substitution in the reverse transcriptase gene is associated with resistance to nucleoside reverse transcriptase i inhibitors (nRTIs). Methods such as MEDS which assume a selective change to a specific amino acid are disadvantaged in these situations. Conversely, swMutSel is designed for situations where there are lineages both before and after the directional selection. In this dataset, there is generally only one sequence for each patient following the use of antiretroviral drugs. As a result, the continued pattern of change that helps to identify the new selective constraints is absent, compromising the accuracy and power of swMutSel.

## Discussion

The first methods for detecting positive selection focused on diversifying selection, such as during pathogen—host co-evolution. The evolutionary dynamics of these situations resulted in a continued elevated rate of amino acid change, which could be detected based on the over-abundance of non-synonymous substitutions. Under these circumstances, it is both difficult and less important to characterize the nature of the changes, as this nature evolves as the interactions between pathogen and host changes.

With the awareness of the importance of episodic directional selection, a few methods have been developed that explicitly model the changes in selective constraints at specific sites. The MEDS approach modifies the PAML model by assuming that there is a shift in the propensity for a single unknown amino acid. The swMutSel method, by contrast, uses a mutation selection model where there is a arbitrary difference in propensities for all of the different amino acids. Given the larger number of adjustable parameters, it is important that the performance be evaluated for both stimulated and real data (Rodrigue 2013).

In agreement with previous results (Tamuri et al. 2009; dos Reis et al. 2011; Tamuri et al. 2012), we find swMutSel to be both effective and conservative. swMutSel does exceptionally well in the situations for which it was designed—when there is information available about the evolutionary dynamics both before and after the change in selection. Under different conditions, such as in the HIV drug resistance data, swMutSel still performs effectively, although not as well as PAML. While PAML was not created to look for directional selection, the results of our tests on drug resistance mutations in HIV indicate it is capable of finding it under certain circumstances, such as when there are numerous sites undergoing convergent changes in multiple lineages. Adding a specific form of directional selection to the PAML model, as in MEDS, seems to decrease the effectiveness, especially when the change in selection is strong.

Of the three methods examined, both swMutSel and PAML have acceptable false positive rates (<$5\%$) in all cases. In contrast, for the simulated data, MEDS generates an extremely high rate of false positives, exceeding 60% in some cases. A possible explanation is that the null model in MEDS assumes that the equilibrium distribution of amino acids is the same at all sites, an assumption that is increasingly seen as unrealistic. MEDs false positive rate seems to increase with the length of the branches. This suggests why MEDS does not generate an excessive number of false positives in the HIV drug resistance analysis—the episodic directional selection occurs at the tips of the branches.

## Methods

### Models

Most models of detecting molecular positive selection are based on comparing the number or rate of non-synonymous changes to that of synonymous changes. This model is implemented in a number of programs, including the widely used program PAML (Yang 2007). In PAML, the rate matrix *Q* for substitutions between the various codons are calculated using

1 $$q_{{ij}} = \left\{ {\begin{array}{*{20}l} {0,} \hfill & {{\text{multiple changes in a codon}},} \hfill \\ {\pi _{j} ,} \hfill & {{\text{synonymous transversions}},} \hfill \\ {\kappa \pi _{j} ,} \hfill & {{\text{synonymous transitions}},} \hfill \\ {\omega \pi _{j} ,} \hfill & {{\text{non-synonymous transversions}},} \hfill \\ {\omega \kappa \pi _{j} ,} \hfill & {{\text{non-synonymous transitions}}} \hfill \\ \end{array} } \right.{\text{ }}$$

where $\kappa$ is the transition/transversion ratio, $\pi _{j}$ is the equilibrium frequency of codon *j* and $\omega = \frac{d_{N}}{d_{S}}$, the ratio of the rates of non-synonymous and synonymous substitutions (Nielsen and Yang 1998). A value of $\omega > 1$ indicates positive selection is occurring along the sequence, while $\omega < 1$ indicates purifying selection. In order to demonstrate positive selection, the program calculates the likelihood of the observed sequences resulting from two models of evolution, only one of which allows for the possibility of $\omega > 1$. The appropriate likelihood ratio test (LRT) is used to decide if there is significant support for some locations evolving under positive selection. In such a case, the Bayes empirical Bayes test (Yang et al. 2005) can be used to estimate which sites are undergoing positive selection.

As discussed in the introduction,* d*_N_/*d*_S_ is expected to be useful for diversifying selection, but its accuracy and power for detecting directional selection is unclear, an important consideration as directional selection is perhaps more common during evolution (Hughes 2007). The HyPhy package (Pond et al. 2005) includes a model for directional selection (MEDS) which has been tested on drug resistance data for HIV sequences with some success (Murrell et al. 2012). For this method, the substitution rate is modeled similarly to Eq. 1 except the nucleotide rate parameters are in a more general form and are a function of the nucleotide frequencies rather than the codon frequencies, a modification that yields less bias in * d*_N_/*d*_S_ estimates (Spielman 2015). In addition, directional selection is represented by a weighting term $\omega _T$ that increases the rate of substitutions to a target amino acid *T* and decreases the rate of substitutions away from this target

2 $$q_{{ij}} = \left\{ {\begin{array}{*{20}l} {0,} \hfill & {{\text{multiple changes in codon}},} \hfill \\ {\theta (i,j)\alpha \pi _{{j,k}} ,} \hfill & {AA(i) = AA(j)} \hfill \\ {\theta (i,j)\beta \pi _{{j,k}} ,} \hfill & {AA(i) \ne T,AA(j) \ne T,} \hfill \\ {} \hfill & {\;\;\;\;\;\;\;\;\;AA(i) \ne AA(j)} \hfill \\ {\theta (i,j)\beta \pi _{{j,k}} \omega _{T} ,} \hfill & {AA(i) \ne T,AA(j) = T} \hfill \\ {\theta (i,j)\beta \pi _{{j,k}}/ \omega _{T} ,} \hfill & {AA(i) = T,AA(j) \ne T} \hfill \\ \end{array} } \right.{\text{ }}$$

where $\theta\, (i,j)$ is the exchangeability between codons *i* and *j*, $\pi _{j,k}$ represents the equilibrium frequency of the nucleotide at position *k* of codon *j*, where *k* indicates the position of the base substitution, and *AA*(*i*) is the amino acid encoded by codon *i*. The statistical support for a value of $\omega _T > 1$ indicates the presence of directional selection. As the target amino acid is not known a priori, all twenty amino acids are considered, with the maximum difference in likelihood used to identify the most likely target residue. Note that the implementation based on a target amino acid represents a special case of directional selection where the selection shifts towards a specific amino acid (rather than a number of possible amino acids), and cannot represent relaxation of selective constraints.

Tamuri, dos Reis, and Goldstein developed a different sitewise codon based mutation selection model (swMutSel) for detecting changes in selective constraints (Tamuri et al. 2014) based on the codon models introduced by (Halpern and Bruno 1998). (swMutSel is available from https://github.com/tamuri/swmutsel.) In this model, the rate of substitution of codon *i* to codon *j* is equal to the product of a mutation rate based on the Hasegawa, Kishino and Yano (HKY) substitution model (Hasegawa et al. 1985) and the fixation probability derived by Kimura for the corresponding change of amino acid  (Kimura 1983). For a change of a single base in the codon at position *k* in the sequence,

3 $$q_{ij}^k =\nu \kappa ^{n}\pi _{j,k}\frac{S_{j,i,k}}{1-e^{-(S_{j,i,k})}}$$

where $\nu$ is a scaling factor, $\kappa$ is the transition/transversion ratio, *n* is one or zero depending upon whether the base change is a transition or transversion, and scaled selection coefficient $S_{j,i,k} = 2 N_e \left( m_{j,k} - m_{i,k} \right)$, where $m_{i,k}$ and $m_{j,k}$ are the Malthusian fitnesses of the amino acids corresponding to codons *i* and *j* in position *k*, respectively. The rate of substitution between codons that differ by more than one base is assumed to be zero. Unlike other methods which have one *Q* matrix for the entire sequence, swMutSel has a *Q* matrix for each site, requiring 19 adjustable parameters per site [$\{F_{i,k}\}$ for each amino acid, where one value can be set to zero], in addition to the base frequencies, transition/transversion ratio, and tree branch lengths which are the same for all sites. This high level of parametrization requires a much higher amount of data than other methods, which limits this approach to situations were many sequences are available. The use of the likelihood ratio test automatically accounts for the additional adjustable parameters for the model representing a change in selection.

PAML, MEDs, and swMutSel are all able to model episodic changes of selection. In PAML and MEDS, one can specify a set of branches (the *foreground branches*) that are allowed to evolve with some locations characterised by $\omega > 1$ or $\omega _T > 1$. This would be appropriate if diversifying selection occurred on only part of the tree, or directional selection induced a burst of non-synonymous substitutions along a specific branch or clade. swMutSel attempts to detect directional selection by modeling the evolution on both sides of the shift in conditions, and seeing whether there is support for a a corresponding change in selective constraints. In swMutSel, one first assumes as a null hypothesis that there is no change of selection in the tree, so the data is fit to a single set of rate matrices of the form of Eq. 3. The tree is the divided up into different parts corresponding to different evolutionary contexts, for instance, between a virus evolving in different hosts, and the sequence data is modeled by a set of rate matrices, where each location *k* in each context is assigned to a different rate matrix. Each site is evaluated independently using the likelihood ratio test or parametric bootstrapping to determine if the data at that site justifies rejection of the null hypothesis of no change in selection. A change in selection is interpreted as directional selection at that location. Given the flexibility of the rate matrix of Eq. 3, the change can be either in the magnitude of selection (relaxed selection, increased selection) or the nature of the constraints (e.g., preference for hydrophylic residues changing to a preference for aromatic residues).

### Comparisons with Simulated Data

The tests of PAML, MEDS, and swMutSel were performed on the results of simulations of a 500 codon length protein based on Eq. 3 as modeled in swMutSel (Tamuri et al. 2012). The majority of locations were simulated using site-specific parameters estimated for two sets of proteins, a secreted alkaliphilic lipase estA (example: embl DQ250714) and DNA binding protein (embl CAG40984). The simulations were performed with symmetric bifurcating trees of 16 and 256 taxa of the type shown in Fig. 1, where every branch was the same length *d*. A change in selective constraints representing directional selection occurred at a fixed fraction of sites ($\theta$) at the midpoint of the branch connecting one fourth of the taxa to the rest of the tree. The simulations were analyzed using the three different approaches. PAML and MEDS incorporate tree branch-length optimization procedures as part of their analysis. The swMutSel analysis used estimated branch-lengths as derived from PAML.

### Comparison with HIV Drug Resistance Data

HIV drug resistance data was taken from (Murrell et al. 2012) which was originally tested on the MEDS method included in HyPhy. These data consist of sequences from three different proteins. The first set contains 122 protease sequences, the second contains 295 integrase sequences, and the third contains 476 reverse transcriptase sequences. Each of these datasets have an associated phylogenetic tree used in the paper with foreground branches labelled. The branch lengths of these trees have been re-estimated using the various methods, and the same foreground branches have been used for testing both the branch-site model of PAML and swMutSel.

## Electronic supplementary material

Below is the link to the electronic supplementary material.

Supplementary material 1 (TXT 3  kb)

Supplementary material 2 (PDF 68 kb)
