# Supplementary material for: Finding Direction in the Search for Selection
Source: J Mol Evol. 2016 Dec 2;84(1):39–50. doi: 10.1007/s00239-016-9765-5 (PMC5253163; doi:10.1007/s00239-016-9765-5)
Supplement: Supplementary file 2 — Supplementary material 2 (PDF 68 kb) [file 239_2016_9765_MOESM2_ESM.pdf]

Finding direction in the search for selection

Journal of Molecular Evolution

Grant Thiltgen, Mario dos Reis and Richard A. Goldstein
Corresponding Author: R. A. Goldstein, Division of Infection & Immunity, University College London
Tel.: +44 (0)20 3108 2206
Email: r.goldstein@ucl.ac.uk

$\theta = 0.01$

	PAML				MEDS				swMutSel			
d	Sens	FPR	MC	AUC	Sens	FPR	MC	AUC	Sens	FPR	MC	AUC
$S = 0.1$												
0.01	0	0	0	0.493	0	0.002	-0.004	0.482	0	0	0	0.5
0.1	0	0	0	0.5	0	0.059	-0.025	0.533	0	0	0	0.5
0.2	0	0	0	0.5	0.400	0.115	0.088	0.682	0	0	0	0.5
0.5	0	0	0	0.499	0.800	0.305	0.106	0.839	0	0	0	0.308
1	0	0.010	-0.010	0.484	1	0.509	0.098	0.977	0	0	0	0.317
$S = 0.5$												
0.01	0	0	0	0.498	0	0.010	-0.010	0.462	0	0	0	0.497
0.1	0	0	0	0.5	0	0.075	-0.028	0.610	0	0	0	0.495
0.2	0	0	0	0.5	0.400	0.107	0.093	0.745	0	0	0	0.501
0.5	0	0	0	0.475	0.600	0.307	0.063	0.679	0	0	0	0.686
1	0	0	0	0.492	1	0.602	0.081	0.963	0.200	0	0.445	0.843
$S = 1.0$												
0.01	0	0	0	0.499	0	0.008	-0.009	0.366	0	0	0	0.5
0.1	0	0.004	-0.006	0.499	0	0.042	-0.021	0.510	0	0	0	0.5
0.2	0	0	0	0.5	0.200	0.131	0.020	0.480	0.200	0	0.445	0.686
0.5	0	0	0	0.493	0.800	0.311	0.105	0.724	0	0	0	0.997
1	0	0.004	-0.006	0.961	1	0.552	0.090	0.972	1	0	1	1
$S = 5.0$												
0.01	0	0	0	0.496	0	0.004	-0.006	0.777	0	0	0	0.799
0.1	0	0	0	0.5	0.200	0.059	0.059	0.545	1	0	1	1
0.2	0	0	0	0.5	0	0.087	-0.031	0.338	1	0	1	1
0.5	0	0	0	0.499	0	0.299	-0.065	0.104	1	0.002	0.912	1
1	0	0.006	-0.008	0.941	0	0.612	-0.125	0.031	1	0	1	1
$S = 10.0$												
0.01	0	0	0	0.5	0	0.004	-0.006	0.695	0.400	0	0.631	1
0.1	0	0	0	0.5	0	0.063	-0.026	0.427	1	0	1	1
0.2	0	0	0	0.5	0	0.109	-0.035	0.243	1	0	1	1
0.5	0	0	0	0.495	0	0.331	-0.070	0.092	1	0.002	0.912	1
1	0	0	0	0.487	0.200	0.596	-0.080	0.187	1	0	1	1

Supplemental Table S1: Performance of PAML, MEDS, and swMutSel on simulated data, as measured by sensitivity (Sens), false positive rate (FPR), Matthews Correlation Coefficient (MC), and the area under the curve of the ROC plot (AUC). Data is represented in Figures 3, 4, 5, and 7.

$\theta = 0.05$

	PAML				MEDS				swMutSel			
d	Sens	FPR	MC	AUC	Sens	FPR	MC	AUC	Sens	FPR	MC	AUC
						$S = 0.1$						
0.01	0	0	0	0.5	0	0.019	-0.031	0.469	0	0	0	0.493
0.1	0	0	0	0.5	0.040	0.061	-0.019	0.495	0	0	0	0.499
0.2	0	0	0	0.5	0.280	0.120	0.104	0.638	0	0	0	0.5
0.5	0	0	0	0.494	0.880	0.368	0.228	0.879	0	0	0	0.498
1	0	0.011	-0.023	0.522	1	0.560	0.194	0.991	0	0	0	0.388
						$S = 0.5$						
0.01	0	0	0	0.5	0	0.013	-0.025	0.525	0	0	0	0.497
0.1	0	0	0	0.5	0.120	0.044	0.077	0.598	0	0	0	0.5
0.2	0	0	0	0.5	0.320	0.080	0.181	0.692	0	0	0	0.512
0.5	0	0	0	0.5	0.880	0.309	0.263	0.852	0	0	0	0.682
1	0	0.004	-0.015	0.481	1	0.549	0.198	0.975	0.040	0	0.195	0.849
						$S = 1.0$						
0.01	0	0	0	0.5	0	0.015	-0.027	0.543	0	0	0	0.519
0.1	0	0	0	0.5	0.280	0.055	0.198	0.638	0.040	0	0.195	0.665
0.2	0	0	0	0.498	0.320	0.118	0.132	0.647	0	0	0	0.580
0.5	0	0.004	-0.015	0.489	0.720	0.307	0.192	0.788	0.440	0	0.654	0.977
1	0	0	0	0.853	1	0.577	0.188	0.859	1	0.004	0.960	0.999
						$S = 5.0$						
0.01	0	0	0	0.499	0	0.008	-0.021	0.617	0	0	0	0.916
0.1	0	0	0	0.946	0.120	0.059	0.055	0.530	0.920	0.002	0.936	0.978
0.2	0	0	0	0.5	0	0.109	-0.078	0.291	1	0	1	1
0.5	0	0	0	0.498	0	0.291	-0.142	0.115	1	0	1	1
1	0	0.011	-0.023	0.462	0.080	0.554	-0.207	0.079	1	0.002	0.980	1
						$S = 10.0$						
0.01	0	0	0	0.5	0.240	0.008	0.361	0.787	0.760	0	0.866	0.999
0.1	0	0	0	0.996	0.080	0.063	0.015	0.468	1	0	1	1
0.2	0	0	0	0.981	0	0.076	-0.064	0.263	1	0.002	0.980	1
0.5	0	0	0	0.499	0	0.337	-0.157	0.085	1	0	1	1
1	0	0	0	0.485	0	0.568	-0.249	0.055	1	0.002	0.980	1

Supplementary Table S1 continued

$\theta = 0.10$

	PAML				MEDS				swMutSel			
d	Sens	FPR	MC	AUC	Sens	FPR	MC	AUC	Sens	FPR	MC	AUC
					$S = 0.1$							
0.01	0	0	0	0.5	0	0.009	-0.030	0.497	0	0	0	0.497
0.1	0	0	0	0.499	0.120	0.071	0.055	0.584	0	0	0	0.494
0.2	0	0	0	0.5	0.200	0.073	0.136	0.604	0	0	0	0.488
0.5	0	0	0	0.5	0.700	0.322	0.236	0.792	0	0	0	0.499
1	0	0	0	0.498	0.980	0.636	0.220	0.953	0	0	0	0.285
					$S = 0.5$							
0.01	0	0	0	0.499	0.020	0.011	0.024	0.482	0	0	0	0.5
0.1	0	0	0	0.499	0.160	0.064	0.110	0.606	0	0	0	0.566
0.2	0	0	0	0.497	0.060	0.120	-0.057	0.530	0	0	0	0.521
0.5	0	0	0	0.494	0.700	0.358	0.210	0.751	0	0	0	0.801
1	0	0	0	0.490	1	0.582	0.259	0.947	0.060	0	0.233	0.953
					$S = 1.0$							
0.01	0	0	0	0.5	0	0.016	-0.040	0.516	0	0	0	0.499
0.1	0	0	0	0.5	0.160	0.064	0.110	0.650	0	0	0	0.724
0.2	0	0	0	0.499	0.160	0.102	0.056	0.569	0.020	0	0.134	0.792
0.5	0	0.002	-0.015	0.491	0.600	0.349	0.156	0.666	0.780	0.002	0.860	0.997
1	0	0	0	0.981	1	0.593	0.253	0.824	0.980	0.002	0.978	1
					$S = 5.0$							
0.01	0	0	0	0.5	0.020	0.013	0.017	0.527	0.160	0.002	0.356	0.939
0.1	0	0	0	0.986	0.220	0.069	0.163	0.624	0.800	0.004	0.860	0.967
0.2	0	0	0	0.998	0.020	0.113	-0.092	0.297	1	0.002	0.989	1
0.5	0	0	0	0.984	0	0.302	-0.204	0.109	1	0.002	0.989	1
1	0	0	0	0.491	0.020	0.604	-0.352	0.047	1	0.004	0.978	1
					$S = 10.0$							
0.01	0	0	0	0.499	0.220	0.002	0.427	0.793	0.760	0	0.860	0.978
0.1	0	0	0	0.995	0.020	0.033	-0.023	0.441	0.980	0.004	0.967	1
0.2	0	0	0	0.993	0	0.142	-0.128	0.256	1	0.002	0.989	1
0.5	0	0	0	0.982	0	0.304	-0.205	0.084	1	0.007	0.968	1
1	0	0	0	1	0.020	0.589	-0.342	0.046	1	0.011	0.948	1

Supplementary Table S1 continued

$\theta = 0.20$

d	PAML Sens	PAML FPR	PAML MC	PAML AUC	MEDS Sens	MEDS FPR	MEDS MC	MEDS AUC	swMutSel Sens	swMutSel FPR	swMutSel MC	swMutSel AUC
$S = 0.1$												
0.01	0	0	0	0.5	0	0.010	-0.045	0.479	0	0	0	0.5
0.1	0	0	0	0.5	0.070	0.083	-0.018	0.530	0	0	0	0.5
0.2	0	0	0	0.495	0.110	0.110	0	0.488	0	0	0	0.5
0.5	0	0	0	0.486	0.610	0.355	0.208	0.654	0	0	0	0.5
1	0	0.008	-0.039	0.475	0.990	0.610	0.328	0.860	0	0	0	0.478
$S = 0.5$												
0.01	0	0	0	0.5	0.010	0.018	-0.024	0.483	0	0	0	0.5
0.1	0	0	0	0.5	0.060	0.073	-0.020	0.535	0	0	0	0.508
0.2	0	0	0	0.496	0.130	0.153	-0.025	0.488	0	0	0	0.542
0.5	0	0	0	0.640	0.520	0.368	0.125	0.590	0.030	0	0.155	0.801
1	0	0	0	0.892	0.960	0.620	0.294	0.826	0.200	0	0.408	0.890
$S = 1.0$												
0.01	0	0	0	0.495	0	0.018	-0.060	0.459	0	0	0	0.515
0.1	0	0	0	0.5	0.060	0.088	-0.040	0.534	0	0	0	0.769
0.2	0	0	0	0.5	0.100	0.125	-0.031	0.512	0.030	0	0.155	0.817
0.5	0	0	0	0.974	0.320	0.350	-0.025	0.504	0.820	0.003	0.879	0.995
1	0	0	0	0.890	0.870	0.633	0.204	0.679	1	0.015	0.964	0.999
$S = 5.0$												
0.01	0	0	0	0.5	0.030	0.010	0.068	0.449	0.150	0	0.352	0.927
0.1	0.950	0.030	0.897	0.996	0.130	0.063	0.102	0.539	0.760	0.005	0.832	0.958
0.2	1	0.028	0.936	0.986	0	0.130	-0.170	0.270	1	0.005	0.988	1
0.5	0	0	0	0.997	0	0.355	-0.315	0.091	1	0.008	0.982	1
1	0	0	0	0.997	0.010	0.583	-0.459	0.037	1	0.008	0.982	1
$S = 10.0$												
0.01	0	0	0	0.5	0.100	0.010	0.218	0.662	0.820	0.005	0.872	0.998
0.1	1	0.048	0.895	0.998	0	0.055	-0.107	0.365	1	0	1	1
0.2	0	0	0	0.987	0	0.113	-0.157	0.239	1	0.010	0.976	1
0.5	0	0	0	0.997	0	0.383	-0.332	0.090	1	0.018	0.958	1
1	0	0	0	1	0.040	0.570	-0.425	0.065	1	0.008	0.982	1

Supplementary Table S1 continued

Site	swMutSel	PAML	MEDS
10*	√	√	
12	√		√
13			√
20*	√	√	
35			√
54*	√	√	
60*			√
61			√
63*	√		
71*	√	√	
74*			√
82*	√		
84*			√
90*	√	√	√
93*			√

Supplemental Table S 2: Sites found under MEDS, PAML and swMutSel for the protease dataset. Sites with stars are sites known to have drug resistance mutations.

Site	swMutSel	PAML	MEDS
72			√
97*	√	√	√
140*	√	√	√
143*	√	√	√
148*	√	√	√
155*	√	√	√

Supplemental Table S 3: Sites found under MEDS, PAML and swMutSel for the integrase dataset. Sites with stars are sites known to have drug resistance mutations.

Site	swMutSel	PAML	MEDS
6	√		
41*	√		√
64		√	√
65*	√	√	
67*	√	√	
70*	√	√	
74*		√	
75*	√	√	
98*			√
100*	√	√	√
101*		√	
103*	√	√	√
104			√
116*			√
123	√		
135	√		
151*	√	√	√
165	√		√
181*	√	√	√
184*	√	√	√
188*	√	√	√
190*	√	√	√
215*		√	√
219*		√	
225*		√	
228	√	√	√
230*			√
286			√

Supplemental Table S4: Sites found under MEDS, PAML and swMutSel for the reverse transcriptase dataset. Sites with stars are sites known to have drug resistance mutations.
